# Prevalence of Delayed Discharge Among Patients Admitted to the Internal Medicine Wards: A Cross-Sectional Study

**DOI:** 10.3390/nursrep15030098

**Published:** 2025-03-14

**Authors:** Filippo Binda, Federica Marelli, Valeria Cesana, Veronica Rossi, Nadia Boasi, Maura Lusignani

**Affiliations:** 1 Department of Healthcare Professions, Fondazione IRCCS Ca’ Granda Ospedale Maggiore Policlinico, Via Francesco Sforza 35, 20122 Milan, Italy; federica.marelli@policlinico.mi.it (F.M.); veronica.rossi@policlinico.mi.it (V.R.); 2 Department of Healthcare Professions, Fondazione IRCCS San Gerardo dei Tintori, Via Giovanbattista Pergolesi 33, 20900 Monza, Italy; valeria.cesana@irccs-sangerardo.it; 3 Department of Internal Medicine, Fondazione IRCCS Ca’ Granda Ospedale Maggiore Policlinico, Via Francesco Sforza 35, 20122 Milan, Italy; nadia.boasi@policlinico.mi.it; 4 Department of Biomedical Sciences for Health, University of Milan, Via Carlo Pascal 36, 20133 Milan, Italy; maura.lusignani@unimi.it

**Keywords:** bed-blocking, patient discharge, length of stay, internal medicine

## Abstract

**Background/Objectives**: Hospital bed shortage is a widespread issue affecting healthcare systems globally, often exacerbated by bed-blocking, a phenomenon where patients remain hospitalized longer than medically necessary due to discharge delays. The aim of this study was to evaluate the prevalence of patients with bed-blocker status admitted to the internal medicine wards. **Methods**: This cross-sectional study was conducted at an academic tertiary-level hospital in Milan (Italy) from 1 January to 31 December 2023. All adult patients identified as ready for discharge by the bed management service, but whose actual discharge was delayed by more than 24 h, were included. Clinical data were retrieved from electronic medical and nursing records. This study adhered to the Strengthening the Reporting of Observational Studies in Epidemiology (STROBE) guidelines. **Results**: Out of 2480 admissions to the internal medicine wards, 147 patients (5.9%) experienced delayed discharge. The median hospital length of stay was 22 days (IQR: 15.0–33.0); the median duration of appropriate stay was 6 days (IQR: 2.0–13.0), and the median length of delayed stay was 14 days (IQR: 7.0–21.0). Waiting for transfer to lower-intensity care facilities was the primary cause of delayed discharge. Complications during delayed stays included delirium (31.3%) and hospital-acquired infections (35.4%), particularly urinary tract infections (17.7%). Logistic regression identified older age (≥75 years), extended hospital length of stay, emergency admissions, and discharge to long-term care as independent predictors of bed-blocker status. **Conclusions**: This study highlights delayed discharges as a significant issue in internal medicine wards, driven by advanced age, caregiver absence, and high dependency in activities of daily living.

## 1. Introduction

The shortage of hospital beds is a common problem in healthcare systems [1]. One approach to addressing this shortage is by reducing avoidable delays in the discharge process, thereby minimizing the number of patients occupying a bed without a clinical indication [2]. The phenomenon of bed-blocking, where patients occupy hospital beds longer than medically necessary due to discharge delays, has become a significant concern in internal medicine wards worldwide [3]. Delayed discharges contribute to a bottleneck in hospital operations, resulting in overcrowded wards, delayed admissions from the Emergency Department (ED), and strained healthcare resources [4]. This issue is critical in internal medicine wards, where efficient patient turnover is essential for maintaining continuity of care for acutely ill patients [5].

The prevalence of this problem varies significantly across studies, depending on the context, the definition of bed-blocker, and the chosen study design. A systematic review that included 64 studies conducted primarily in Europe, Canada, and the United States between 1990 and 2014 reported prevalence rates ranging from 1.6% to 91.3% (weighted mean: 22.8%) [6]. The wide variation in prevalence of delayed discharges is also observed within the same country, with a range of 58.4% in the United Kingdom, 43.0% in Spain, 49.7% in Italy, 70.3% in Canada, and 56.8% in the Netherlands [6]. The United Kingdom has been the most productive in studying and publishing research on bed-blocking [7]: recent data from that country highlight that in hospitals, one in six patients is medically fit for discharge; however, due to several factors, their hospital stay is prolonged [8].

The causes of the bed-blocking phenomenon are multifaceted, involving both individual and organizational factors [9,10]. Individual factors include advanced age, frailty, chronic illnesses, cognitive decline, and psychiatric disorders [11,12,13,14]. Additionally, factors such as living alone [15], the absence of a caregiver [16], and social isolation further increase the risk of delayed discharge [17]. Organizational factors, on the other hand, include systemic inefficiencies, such as inadequate discharge planning [18], and shortages in community care facilities [19].

Patients whose discharge is delayed are also exposed to iatrogenic complications and adverse events due to prolonged hospitalization [20], including accelerated functional decline [21], delirium [22], pressure ulcers [23], nosocomial infections [24], and falls [25], all of which lead to prolonged hospital stays. Moreover, several systematic reviews and meta-analyses have reported that the hospitalization of older patients can lead to a decline in long-term health and functioning, potentially resulting in further morbidity and disability [26,27,28,29].

In recent years, Italy has seen a consistent decline in hospital bed availability, largely due to budget constraints and spending cuts [30,31]. This trend had a significant impact during the COVID-19 emergency, when the health system was placed under severe strain because of reduced hospital capacity and underinvestment in community-based care [32]. Consequently, optimizing bed utilization became crucial to meet healthcare needs despite constrained resources. In this context, evaluating and quantifying the issue of delayed discharge is particularly relevant at the hospital level, especially in internal medicine wards. These wards play a pivotal role in hospital bed availability because they admit a significant proportion of patients from the ED [33]. Therefore, the effective management of internal medicine beds is essential for ensuring the overall flow of patients and enhancing the efficiency of the entire hospital.

Given the above, and considering the limited post-pandemic research on this topic conducted in Italy to date, studying delayed discharges in this specific setting is essential to improve the quality of care and ensure the effective use of hospital resources.

The primary aim of this study is to evaluate the prevalence of patients with bed-blocker status admitted to the internal medicine wards of an academic tertiary-level hospital in Italy. The secondary aims are to analyze the clinical patients’ characteristics, complications, and the principal factors associated with the delayed discharge of patients.

## 2. Materials and Methods

### 2.1. Study Design and Setting

This cross-sectional study was conducted at the Fondazione IRCCS Ca’ Granda Ospedale Maggiore Policlinico, an academic tertiary-level hospital in Milan (Italy), between 1 January and 31 December 2023. This major multi-specialty teaching hospital, currently comprising approximately 900 inpatient beds (81 of which are allocated to internal medicine wards) and managing around 46,000 admissions annually, provides comprehensive medical care across various disciplines.

The study population included all adult patients admitted to the internal medicine wards and identified as ready for discharge by the bed management service, but whose actual discharge was delayed by more than 24 h. Patients who did not experience a problematic delay in their discharge were excluded, as were those admitted under a non-ordinary regimen (such as a day hospital) or patients discharged to other hospitals.

Patients admitted to internal medicine wards are predominantly referred from the ED and present with complex health conditions, such as multiple chronic comorbidities, malnutrition, cognitive impairment, and polypharmacy [34]. The most treated pathologies include cardiovascular and metabolic disorders, as well as respiratory diseases.

The study was approved (approval number 3883_2024) by the local Ethics Committee (Comitato Etico Territoriale Lombardia 3). Written informed consent was waived because of the retrospective nature of the study. We estimated a prevalence of 9.5% for patients whose discharge was delayed, based on the largest observational study conducted in Italy published to date [35]. Accordingly, a sample size of 133 patients was deemed necessary to estimate this prevalence with a 95% confidence interval and a margin of error of 5%.

### 2.2. Data Collection

For this retrospective study, data were retrieved from electronic medical and nursing records, and collected using the REDCap electronic data capture tool (version 14.3.13) [36]. Sociodemographic data included age, sex, citizenship, presence of a language barrier, presence of a caregiver and/or legal administrator, and support from social services. Other clinical information, such as hospital admission, major diagnosis category, discharge setting (long-term care center, home, death, or other setting such as nursing homes), and the Blaylock Risk Assessment Screening Score (BRASS) [37], which is used to identify patients at risk for prolonged hospital stays or in need of additional discharge planning, was also recorded.

To define bed-blocker status, the hospital bed management service used the following definition: “An instance where a medically-fit patient is needlessly kept in hospital due to internal organizational/operational factors or where a patient is flagged as in need of alternate level of care and is delayed because of deferred transition of care and/or lack of external transfer-of-care arrangements” [3]. The total length of stay (LOS) in the hospital was divided into two periods: the appropriate stay (from hospital admission to the ready-for-discharge date) and the delayed stay (from the ready-for-discharge date to the actual discharge date).

Finally, to better describe the level of autonomy and social support, the Barthel Index score was recorded to measure functional independence in Activities of Daily Living (ADL) [38], along with the level of social support assessed using the Oslo Social Support Scale (OSSS-3) [39]. Complications that occurred during the prolonged stay, including pressure ulcers, delirium, ground-level falls, nosocomial infections, and immobilization syndrome, were reported as recorded in the electronic nursing charts. The study results were reported following the Strengthening the Reporting of Observational Studies in Epidemiology (STROBE) guidelines [40].

### 2.3. Data Analysis

All data were analyzed using the statistical software Jamovi version 2.3.21 for Macintosh (Jamovi Project, 2025). Descriptive statistics were calculated using frequencies, means, and standard deviations (SD) in order to describe the characteristics of the sample and the periods of hospital stay. For group comparisons, the Fisher’s Exact test was used to evaluate associations between categorical variables, such as the presence of a caregiver and delayed discharge. Continuous variables, including length of stay, were compared using the Mann–Whitney U test due to their non-normal distribution, as indicated by the Shapiro–Wilk test. A multivariate logistic regression analysis was performed to assess the independent predictors of bed-blocker status, with odds ratios (OR) and 95% confidence intervals (CI) being reported. Statistical significance was set at *p* < 0.05 for all tests.

## 3. Results

### 3.1. Descriptive Statistics

During the study period, 147 patients admitted to the internal medicine wards were identified as experiencing delayed discharge. The average age of the patients was 78.2 years (SD 13.6, range: 33–99 years). The majority (95.2%, 140/147) were Italian citizens, while only 3.4% (5/147) had a language barrier. Additionally, 15.0% (22/147) had a legal administrator, and 13.6% (20/147) received support from social services prior to hospitalization. Most patients (82.3%, 121/147) resided in an urban area near the hospital.

The general characteristics of patients are summarized in Table 1.

Overall, the prevalence of patients with delayed discharge was 5.9%, based on a total admission volume of 2480 patients over the course of the year. The total hospital LOS was 4285 days: of these, 1450 days were categorized as appropriate stays, while 2835 days were classified as delayed stays. The median hospital LOS was 22 days (IQR: 15.0–33.0); the median duration of appropriate stay was 6 days (IQR: 2.0–13.0), and the median duration of delayed stay was 14 days (IQR: 7.0–21.0). Delayed stays ranged from 1 to 242 days, with up to 16.3% (24/147) of patients experiencing a delayed stay exceeding 30 days.

The main cause of delayed discharge for 53.7% (79/147) of patients was the waiting time for transfer to lower-intensity care facilities. Fisher’s exact test revealed a statistically significant association between the absence of a caregiver and delayed hospital discharge (*p* < 0.001). Patients without a caregiver experienced a median delayed stay of 20 days (IQR: 9.8–35.5), compared to 13 days (IQR: 7.0–21.0) for those with a caregiver. Among patients without a caregiver, 75% (15/20) were also found to have inadequate social support, as indicated by an OSSS-3 score ≤ 8 points, underscoring that insufficient familiar or community assistance may play a critical role in delaying discharge. Further details regarding the primary causes for delayed discharge are reported in Table 2.

The main complications occurred during the delayed discharge period involved hospital-acquired infections (HAIs), with at least one reported by 35.4% (52/147) of the patients. Among the bloodstream infections, 6.1% (9/147) were caused by multidrug-resistant (MDR) pathogens. High levels of dependency were recorded, with a Barthel Index ≤ 60 points for 70.1% (103/147) of the patients, particularly for those who developed a new onset pressure ulcer (8.2%, 12/147). No complications were reported in the nursing records for 27.2% (40/147) of the patients. Other information is summarized in Table 3.

Table 4 presents the distribution of discharge settings for patients who experienced delayed discharge. This table underscores the significant proportion of patients requiring continued care in specialized facilities. Long-term care centers were the most common discharge setting, with the largest number of patients identified as high-risk (37.0%, 30/81) with a BRASS score ≥ 20 points also being transferred there. In-hospital death occurred in 8.8% (13/147) of patients, highlighting the severity of illness in a subset of the sample, where delayed discharge might have been influenced by end-of-life care considerations.

### 3.2. Regression Analysis Results

The univariate logistic regression presented in Table 5 reveals that several factors, including age, type of admission, hospital LOS, and discharge setting, are significantly associated with bed-blocker status, while discharge to home significantly reduces the likelihood. Older patients (aged ≥ 75 years) had a notably higher probability of being classified as bed blockers. The OR of 1.83 (95% CI: 1.26–2.66, *p* = 0.002) indicates that these patients are 83% more likely to be bed blockers compared to younger patients.

The multivariate logistic regression analysis identifies some independent predictors of bed-blocker status. Hospital LOS remained a strong predictor, with each additional day in the hospital significantly increasing the likelihood of bed-blocker status (OR: 1.08, 95% CI: 1.06–1.09, *p* < 0.001). ED admission also significantly increased the odds (OR: 7.84, 95% CI: 1.60–38.54, *p* = 0.011), indicating that patients admitted through emergency pathways were more likely to experience delayed discharge. Age ≥ 75 years was another significant factor, with older patients exhibiting more than double the odds of bed-blocker status (OR: 2.10, 95% CI: 1.33–3.31, *p* = 0.001). Furthermore, discharge to long-term care centers was highly predictive (OR: 7.41, 95% CI: 4.93–11.15, *p* < 0.001) of bed-blocker status, reinforcing the substantial impact that difficulties in accessing post-acute care have in significantly prolonging hospitalization.

## 4. Discussion

In this retrospective cross-sectional study, the prevalence of patients admitted to internal medicine wards who experienced delayed discharge was 5.9%. The main findings emphasize that advanced age, absence of a caregiver, and significant dependence in ADLs are primary predictors of delayed discharge. Unsurprisingly, complications increased as the length of discharge delay grew; in particular, delirium and HAIs were the most frequently reported complications during the delayed discharge period.

The prevalence of delayed discharge in this study aligns with previous research that included adult patients discharged from internal medicine wards [20]. Specifically, the prevalence rate can vary significantly depending on the clinical setting. For instance, in general surgery wards, the prevalence is usually lower as patients tend to be younger and present less comorbidities [41]. Another potential explanation could be that surgical wards discharge or transfer patients elsewhere as soon as possible to make their beds available [35]. The substantial variation in the reported prevalence of delayed discharges may ultimately also be attributed to methodological differences in data collection [6], as well as to seasonal fluctuations in hospital admissions, which can lead to underestimates or overestimates of the proportion of delayed discharges in studies conducted over periods shorter than one year [42,43,44].

The clinical characteristics associated with delayed discharge include difficulty with mobility, high dependence in ADL, and poor social support, all of which have been identified as significant predictors of delayed discharge in previous studies [45,46,47]. Older age is linked to increasing comorbidities and functional decline. Notably, functional decline, as evidenced by a Barthel Index ≤ 60 points in most patients, represents a key determinant of complexity in internal medicine inpatients. Other major contributors to patient complexity include individual frailty, polypharmacy, and socioeconomic status [48,49]. In particular, the situation of individuals experiencing homelessness underscores the impact of socioeconomic factors, as they often cannot complete follow-up care, face multiple social challenges upon discharge, and have limited support outside the hospital [50]. Significantly, an association between the absence of a caregiver and delayed discharge has been observed in this study, especially among those with poor social support. Previous research on delayed discharges corroborates these findings, showing that caregiver characteristics remain crucial even after accounting for clinical factors [51]. Furthermore, social isolation is associated with both delayed hospital discharge and the number of days of delay in older patients, highlighting their vulnerability to isolation. Older people are particularly vulnerable to social isolation which has a negative impact on their health and well-being with cost implications for healthcare services [52].

Prolonged hospital LOS has been associated with negative outcomes for patients, including functional decline during hospitalization and an increased risk of complications [53,54]. Among these complications, nosocomial infections (particularly those involving MDR pathogens) were the most common. Specifically, hospital-acquired respiratory and urinary tract infections were observed in 18.4% and 17.7% of the study population, respectively. The increased risk of developing both urinary tract infections and perineal skin maceration, which can lead to pressure ulcers, is related to the use of absorbent incontinence pads, commonly used for bedridden elderly patients [55]. Notably, urinary tract infections are the most common cause of infection in hospitalized elderly women, partly due to anatomical differences [56]. Among frail elderly patients, infections (such as urinary tract infections or pneumonia), metabolic disorders (such as hyponatremia, hypoglycemia, or hypoxemia), and environmental factors (such as hospitalization or ED admissions) are well-established triggers for delirium [57]. In the study population, delirium was observed in 31.3% of patients: this prevalence is likely attributable to prolonged LOS and may also be explained by the significant relationship between hospital-acquired urinary tract infections and delirium reported in the literature [58]. Additionally, patients with delirium experienced worse outcomes than those without delirium, including higher rates of falls and the use of physical restraints [59]. These findings align with the existing literature [60], further emphasizing that prolonged LOS increases susceptibility to infections, impairs functional recovery, and adversely affects patient turnover.

In this study, most patients experience delayed discharge while waiting for an available bed in institutional long-term care facilities. Discharges to lower-intensity care facilities are highly complex and require active patient involvement to coordinate referrals and transportation [61]. A report on long-term care by the European Commission reveals that a significant proportion of older people in need of institutional care are currently on waiting lists [62]. The necessity of waiting for a long-term care bed or nursing homes restricts access for new admissions requiring acute care and reduces overall hospital capacity [63]. Moreover, older adults who are waiting for a place in a nursing home die before they are admitted: the 8.8% in-hospital mortality rate indicates that some delayed discharges may be linked to end-of-life care decisions, highlighting the potential benefits of hospital-based specialist palliative care for improving patients’ quality of life [64].

Efficient discharge planning is an important component of hospital care [65]. This study highlights the value of using screening tools, such as the BRASS score or similar instruments, to identify at an early stage those patients who are unlikely to return directly home after hospitalization [66]. Despite 87.8% of patients in this study being accurately identified as at risk for a difficult discharge, early detection of those not eligible for discharge to home did not reduce inappropriate hospital utilization. Several studies have tried to develop screening instruments to address problems associated with the discharge process in patients hospitalized for acute medical conditions [67,68,69]. However, these tools differ considerably in their outcome measures and do not specifically address the issue of discharge delays related to transfers to long-term care facilities [70]. For this reason, a daily situation report is essential for identifying and addressing potential barriers to discharge, ensuring timeliness and safety through the involvement of a dedicated bed management team [71]. In addition, implementing tracking systems that provide regular updates on patient status and discharge planning can further support efforts to achieve timely discharges [72].

There are several strengths to this study. First, the recruitment of patients over a full year allows for more precise estimates compared to other studies conducted within the Italian context over much shorter periods. Second, a standardized definition was used to classify the condition of bed blocker. Third, data collection and verification were rigorous, supported by monitoring from the bed management team.

However, some limitations should be acknowledged. The principal limitation of this study is that it was conducted in a single hospital, and therefore the findings may not be generalizable to other clinical settings with different healthcare system structures. However, future research may include cross-national or international collaborations to enhance the generalizability of the results. Additionally, when describing complications that arose during the delayed discharge period, only those attributable to prolonged hospitalization were considered. Given the nature of the study, it was not possible to determine whether a particular factor causes a specific outcome, thereby precluding definitive conclusions about the direction of the association. Finally, no distinction was made regarding discharge times between transfers to private and public nursing homes, where patients’ financial resources directly influence availability.

## 5. Conclusions

This study highlights delayed discharges as a significant issue in internal medicine wards, driven by advanced age, high dependency in ADLs, caregiver absence, and limited social support. Prolonged hospital stays increase the risk of complications, contributing to poorer outcomes and inefficient hospital resource utilization. The primary cause of delays was the lack of availability in long-term care facilities, underscoring the need for expanded post-acute care infrastructure. Tools like the BRASS score, combined with daily situation reports and multidisciplinary coordination, could improve discharge planning.

## Figures and Tables

**Table 1:** Sociodemographic and clinical characteristics.

Variables	N = 147	(%)
Sex		
Female	79	53.7%
Male	68	46.3%
Age group (years)		
≤45 years	5	3.4%
46–64 years	15	10.2%
65–74 years	19	12.9%
75–84 years	52	35.4%
≥85 years	56	38.1%
Major diagnosis category		
Infectious	35	23.8%
Neurologic	31	21.1%
Respiratory	21	14.3%
Cardiovascular	11	7.5%
Gastrointestinal	9	6.1%
Psychiatric	8	5.4%
Orthopedic	7	4.8%
Nephrological	4	2.7%
Oncologic	3	2.0%
Other	18	12.3%
Hospital admission		
Emergency	144	98.0%
Planned	3	2.0%
Blaylock Risk Assessment Screening Score		
High risk (≥20 points)	81	55.1%
Moderate risk (11–19 points)	48	32.7%
Low risk (0–10 points)	18	12.2%

**Table 2:** Reasons for delayed discharge in patients admitted to internal medicine wards.

Reasons for Delayed Discharge	N = 147	(%)	Delayed Stay (Days) ^1^
Awaiting place in long-term care center	46	31.3%	11.5 (7.0–21.0)
Home care support not available	35	23.8%	13.0 (7.0–19.0)
Awaiting place in nursing home	21	14.3%	13.0 (8.0–16.0)
Absence of a caregiver	20	13.6%	20.0 (9.8–35.5)
Awaiting place in hospice	12	8.2%	15.5 (7.3–24.8)
Condition of indigence or poverty	11	7.5%	14.0 (5.5–28.5)
Awaiting diagnostic procedures	2	1.4%	9.5 (7.3–11.8)

^1^ Days of delayed stay are reported as median and interquartile range (IQR).

**Table 3:** Complications and need for assistance in care during delayed discharge period.

Variables	N = 147	(%)
Complications occurred during delayed stay		
Delirium	46	31.3%
Urinary tract infection	26	17.7%
COVID-19 infection	20	13.6%
Bloodstream infection	19	12.9%
New-onset pressure ulcer	14	9.5%
Ground-level fall	11	7.5%
Hospital-acquired pneumonia	7	4.8%
Immobilization syndrome	6	4.1%
Barthel Index		
Total dependence (0–20 points)	46	31.3%
Severe dependence (21–60 points)	57	38.8%
Moderate dependence (61–90 points)	25	17.0%
Slight dependence (91–100 points)	19	12.9%
Oslo Social Support Scale		
Poor social support (3–8 points)	66	44.9%
Moderate social support (9–11 points)	77	52.4%
Strong social support (12–14 points)	4	2.7%

**Table 4:** Discharge settings.

Discharge Setting	N = 147	(%)
Long-term care center	59	40.2%
Home care	28	19.1%
Nursing home	25	17.0%
In-hospital death	13	8.8%
Home	11	7.5%
Hospice	8	5.4%
Supportive housing	3	2.0%

**Table 5:** Univariate analyses.

Bed-Blocker Status		Odds Ratio	CI 95% ^1^	*p*-Value
Sex	Female	1.14	0.82–1.59	0.443
Age	(≥75 years)	1.83	1.26–2.66	0.002
Citizenship	Other nationalities	0.96	0.29–3.13	0.951
Hospital admission	Emergency	4.77	1.17–19.43	0.029
Length of hospital stay	(days)	1.07	1.06–1.08	<0.001
Discharge setting	Long-term care center	10.29	7.13–14.86	<0.001
	Nursing home	4.56	2.76–7.53	<0.001
	Home	0.14	0.09–0.20	<0.001

^1^ Confidence Interval 95%.

## Data Availability

The original data of this study are available in the open research repository FigShare at https://doi.org/10.6084/m9.figshare.28194503.v1 (accessed on 12 March 2025).

## Public Involvement Statement

No public involvement in any aspect of this research.

## Guidelines and Standards Statement

This manuscript was drafted in accordance with the STROBE (Strengthening the Reporting of Observational Studies in Epidemiology) guidelines.
